# Magnetic resonance imaging findings in children with intractable epilepsy compared to children with medical responsive epilepsy

**DOI:** 10.22037/ijcn.v16i2.2710

**Published:** 2022-03-14

**Authors:** Azime KHOSRONEJAD, Elham RAHIMIAN, Mohammad RAISZADEH, Shahriar NAJAFIZADE, Alireza RANAIE-KENARSARI, Susan AMIRSALARI

**Affiliations:** 1 **Baqiyatallah University of Medical Sciences, Tehran, Iran**; 2 **Neuroradiologist, Haghighat Neuroimaging Center, Tehran, Iran**; 3 **School of Medicine, Trauma Research Center, Bqiyatallah Al-Azam Hospital, Baqiyatallah University of Medical Sciences, Tehran, Iran**; 4 **Medical student, Baqiyatallah University of Medical Sciences, Tehran, Iran**; 5 **New Hearing Technologies Research Center, Baqiyatallah University of Medical Sciences, Tehran, Iran**

**Keywords:** Intractable epilepsy, Drug-responsive epilepsy, Magnetic Resonance Imaging (MRI)

## Abstract

**Objective:**

Epilepsy is a common brain disorder characterized by a persistent tendency to develop seizures in neurological, cognitive, and psychological contents. Magnetic Resonance Imaging (MRI ) is a neuroimaging test facilitating the detection of structural epileptogenic lesions. This study aimed to compare the MRI findings between patients with intractable and drug-responsive epilepsy.

**Material & Methods:**

This case-control study was conducted from 2007 to 2019. The research population encompassed all 1-16-year-old patients with intractable epilepsy referred to the Shafa Neuroscience Center (n=72) (a case group) and drug-responsive patients referred to the pediatric neurology clinic of Baqiyatallah Hospital (a control group).

**Results:**

There were 72 (23.5%) patients in the intractable epilepsy group and 200 (76.5%) patients in the drug-responsive group. The participants’ mean age was 6.70± 4.13 years, and there were 126 males and 106 females in this study. Normal brain MRI was noticed in 21 (29.16%) patients in the case group and 184 (92.46%) patients in the control group.

Neuronal migration disorder (NMD) was also exhibited in 7 (9.72%) patients in the case group and no patient in the control group. There were hippocampal abnormalities and focal lesions (mass, dysplasia, etc.) in 10 (13.88%) patients in the case group and only 1 (0.05%) patient in the control group.

Gliosis and porencephalic cysts were presented in 3 (4.16%) patients in the case group and no patient in the control group. Cerebral and cerebellar atrophy was revealed in 8 (11.11%) patients in the case group and 4 (2.01%) patients in the control group. Corpus callosum agenesis, hydrocephalus, brain malacia, and developmental cyst were more frequent in the case group; however, the difference between the groups was not significant.

**Conclusion:**

The MRI findings such as hippocampal abnormalities, focal lesions (mass, dysplasia), NMD, porencephalic cysts, gliosis, and atrophy are significantly more frequent in children with intractable epilepsy than in those with drug-responsive epilepsy.

## Introduction

Seizures are attacks of the nervous system, resulting in sudden and reversible changes in mental status or somatosensory function, and tend to have a stereotypical and repetitive nature. Epilepsy is a brain disorder characterized by a persistent tendency to develop seizures in the neurobiological, cognitive, and psychological contexts. It is a well-known disorder of ancient times and dates back to the Stone Age. About 4-5% of children experience at least one seizure (febrile or afebrile) during the first sixteen years, 3% of which are caused by epilepsy (1, 2).

The clinical diagnosis of epilepsy requires at least one unprovoked seizure with a second seizure attack or abnormal EEG and sufficient clinical information to indicate a continuing susceptibility to recurrence.

The duration of epilepsy attacks varies from a few seconds to a few minutes (rarely a few hours), abruptly terminating and gradually returning to baseline. There may be warning signs before the attack, including Aura or decreasing consciousness, and after the attack, called postictal situation (1, 2).

According to international classification, there are two forms of epilepsy: Focal or Generalized epilepsy. In generalized epilepsy, there are different subgroups, including tonic, colonic, tonic-colonic, absence, and others. The differential diagnoses of seizures in children are migraine attacks, transient ischemic attacks, syncope, dizziness, hypoglycemia, respiratory arrest, tics, and hysteria (1-4).

EEG and neuroimaging are the best ways to diagnose epilepsy; however, they must be accompanied with checking history and examinations. MRI is the most valuable imaging, which is more sensitive than CT scanning. Imaging aims to diagnose structural disorders causing seizures (5, 6).

Epileptic drug therapy began with discovering the anticonvulsant effect of potassium bromide in 1854. It was a preferred drug for epilepsy treatment for up to 70 years. Phenobarbital was then discovered and synthesized in 1912. Twenty-five years after the advent of Phenobarbital, Phenytoin and then Trimethadione were introduced in 1938. Carbamazepine was introduced in 1967, followed by Valproic acid in 1974 (4, 7).

After fifteen years, new anticonvulsants (namely Lamotrigine and Gabapentin) were introduced, and many new drugs were then introduced a few years later. The new generation of antiepileptic drugs is often used to treat refractory seizures (7, 8).

In 70% of cases, epileptic seizures are completely controlled with one drug. In 10% of the cases, the severity and frequency of attacks decrease, and in 20% of such patients, seizures remain uncontrollable and resistant despite appropriate pharmacological treatment (9, 10).

Although there has been significant progress in the treatment of epilepsy during the last few decades, a highly remarkable percentage of patients resist drug therapy. Studying intractable epilepsy is important since the treatment is complicated.

According to the published literature on the significance of the MRI imaging in epileptic patients, there should be investigations to detect whether it is helpful in the differential diagnosis of refractory epilepsy and drug-responsive epilepsy (5). Accordingly, the present study aimed to compare the MRI findings of patients with intractable and drug-responsive epilepsy.

## Materials&Methods

 This case-control study was conducted from 2007 to 2019. The research population encompassed all 1-16-year-old patients with intractable epilepsy referred to Shafa Neuroscience Center (n=72 patients) (a case group) and drug-responsive patients referred to the pediatric neurology clinic of Baqiyatallah Hospital (a control group).

Intractable epilepsy is defined as having at least one seizure per month despite taking three appropriate conventional antiepileptic drugs. In the control group, children and adolescents were suffering from a type of epilepsy responsive to routine antiepileptic drugs and had no seizure attacks for at least one year after taking one to three antiepileptic drugs.

The required data were extracted from medical records in the archives of Baqiyatallah and Shafa clinics after obtaining parents’ and caregivers’ informed consent. All MRI imaging results were reported by one neuroradiologist to unify the findings.

Research variables were age, gender, and the MRI findings. MRI can evaluate abnormalities such as tumors, arteriovenous malformations, cysts, strokes, focal atrophy, hippocampal atrophy, and other structural abnormalities. Furthermore, signal abnormities and volumetry of the lobes and ventricles were analyzed using the MRI studies. A few variables were partially inserted or omitted in some cases, and this may be considered the limitation of the study in completing the findings. Moreover, in a few cases, calling the parents to complete the information defects was not possible. 

Statistical analysis was performed with SPSS software version 17(Chicago, IL, USA). Kolmogorov-Smirnov test was performed to examine the normal distribution of the data. Then an Analysis of Variance (ANOVA) and Fisher's exact tests were used to investigate the relationship among the research variables. Statistically, the significance level was set to be *p* <0.05 in this study.

## Results

Patients

The patients were studied in two groups (namely medical responsive epilepsy and intractable epilepsy) from July 2007 to August 2019. Seventy-two (23.5%) patients were included in the intractable epilepsy group, and 200 (76.5%) patients were in the drug-responsive group. One case in drug-responsive was excluded from the study due to refusing to cooperate in the study. 

The participants’ mean age was 6.70± 4.13. Table 1 presents the mean age and gender of each group. In this study, there were 126 males and 106 females. The frequency of the male gender was higher than the female; however, the difference was not significant. Finally, the analysis of age and gender revealed no significant difference between the two groups and also between cases with normal and abnormal MRI findings in the two groups.

MRI Findings

Table 2 shows the MRI findings in both groups. Normal brain MRI was noticed in 21(29.16%) patients in the case group and 184 (92.46%) patients in the control group.

Neuronal migration disorder (NMD) refers to a group of cerebral dysgenesis occurring during neurogenesis in the fetus. NMD was diagnosed in seven cases (9.72%), all of whom were assigned to the intractable epilepsy group. 

Hippocampal abnormalities (atrophy, sclerosis, rounding, and displacement) were noticed in 10 (13.88%) patients in the case group and only 1 (0.05%) case in the control group (*p*<0.001). Focal lesions (namely mass, dysplasia ) were observed in 10 (13.88%) patients in the case group and only 1 (0.05%) case in the control group (*p*<0.001).

The frequencies of global, hemispheric, and cerebellar atrophy were significantly higher (*p*=0.003) in the intractable epilepsy group (11.11% vs. 2.1%).

Signal intensities were higher in the brain white matter of the drug-responsive group than in the intractable epilepsy group (3.01% vs. 0.05%); however, the difference was not statistically significant (*p* = 0.679).

Findings such as developmental cyst, hydrocephalus, brain malacia, and corpus callosum agenesis were insignificantly more frequent in the case group. However, porencephalic cyst and gliosis were significantly higher in the case group (Table 2).

## Discussion

Almost all patients with the new-onset episodes of epilepsy must be evaluated with imaging for an abnormality in the brain structure and anatomy. MRI is the best diagnostic instrument to evaluate abnormalities such as tumors, arteriovenous malformations, atrophies, cysts, and others. However, MRI can calculate the volume of different brain parts by using software and performing calculations on images (11) as such it can reveal brain malacia, atrophy, ventricular dilation, and others. However, this is documented that many abnormal MRI findings have a significant association with different types of epilepsy (5). This study aimed to compare the MRI findings between patients with intractable and drug-responsive epilepsy. 

Neuronal migration disorder refers to a group of disorders with the same ethiopathological mechanism of inability to migrating neuroblasts during neurogenesis. Seizures are one of the NMD symptoms, and the other NMD symptoms are mental retardation, abnormal muscle tone, failure to thrive, and so on. The seizures in NMD are resistant to the AED therapy. In a similar study, out of 202 patients with intractable seizures, 26 (12.9%) patients were diagnosed with NMD, which was higher than our study (9.72%). The seizures in all patients diagnosed with NMD in both studies were resistant to drugs (12). Accordingly, NMD is significantly more frequent in intractable epilepsy (p<0.001). Moreover, in many studies, a significant number of patients with NMD have presented intractable seizures (13, 14).

Abnormalities in the hippocampal area ( i.e., rounding, displacement, etc.) are noticed in congenital malformations. Atrophy in the hippocampal area is normal in old age; however, it is a pathological finding in children. In complex partial seizures, the most common histological finding is mesial temporal sclerosis with reduced hippocampal volume and hippocampal hyperintensities in the T2W FLAIR images. In Mastu Fuji et al.'s study, a significant relationship between status epilepticus and hippocampal anomalies was documented during the MRI studies (15). In other studies, the comparison of hippocampal volumes showed the bigger size of the right hippocampus compared to the left side in the intractable epilepsy group (16, 17). According to the current study, the MRI findings of hippocampal abnormalities in children with epilepsy (e.g., hippocampal atrophy and sclerosis) are more significant in the intractable epilepsy group (*p*<0.001). The temporal lobe is a part of the brain processing sensory signals into derived meanings for many reasons such as the appropriate retention of visual memory, language comprehension, and emotion association (18). In the brain MRI, we mainly used T1W images to evaluate the temporal lobe, T2W images to study all brain parts, and fluid-attenuated inversion recovery (FLAIR) images to investigate the anatomy and signals of the hippocampus and temporal lobes. Furthermore, diffusion-weighted imaging is routinely used in epilepsy workups (19, 20). Temporal lobe epilepsy (TLE) is a chronic neuronal disease with repetitive and focal seizures orienting from the temporal lobe and lasts one or two minutes and can shift to a generalized seizure. TLE is always diagnosed in childhood and adolescence using medical history, laboratory tests, and imaging. However, many studies (e.g., Sureka et al. &Henry et al.) on the atrophy of the temporal lobe have reported the critical role of these findings in epilepsy.

In Moran et al.'s study, the brain volumetry was obtained on 62 cases with TLE and 20 healthy patients, indicating that the patients with TLE have 13.0% lower temporal lobe volume in the MRI studies on average. Further, this study revealed that all patients had intractable epilepsy (21). In their study, 16 patients were diagnosed with TLE, 3 (18.75%) patients had drug-responsive, and 13 (81.25%) patients had intractable epilepsy (22,23). In the present study, the whole temporal lobe atrophy was not reported; however, there was dilation of the temporal horn of the lateral ventricle, which was not significantly different between the two groups (*p*=0.118). Moreover, hippocampal abnormalities were significantly more in the intractable epilepsy group (*p*<0.001). In our study, global, hemispheric, and cerebellar atrophy were higher in the case group (11.11 vs. 2.01, *p*=0.003) (Table 2).

Brain tumors are one of the main etiologies of intractable epilepsy. In another study on epileptic children, about 4% of children had tumoral masses in their brains (5). A similar study on the intractable MRI of children who underwent surgery showed focal masses on 160 (72%) out of 222 cases, and 62 cases (27%) had normal brain MRI (24). In the present study, brain tumors were diagnosed in 8 (11.11%) intractable patients and only one drug-responsive patient as such they were significantly more frequent in the case group. (*p*<0.001). Porencephalic cysts may be due to encephalomalacia in the brain having many etiologies such as brain ischemia, trauma, infections, and intra-parenchymal hemorrhage. In another study, 12 patients diagnosed with porencephalic cysts were investigated, all of whom had intractable epilepsy (25),. In our study, we diagnosed three cases of the porencephalic cyst, and all of the cases were in the intractable group. 

**Table 1:** Participants’ demographic information and MRI findings

P-value	Intractable		Drug-Responsive		
		N		N	
0.683	7.81±4.02	72	6.30±4.11	199	Age
0.969	33:28	72	93:78	199	Gender (M: F)
0.189	8.03±3.71	52	6.42±4.09	186	Age of Normal MRI
0.907	26:22	52	83:73	186	Gender of Normal MRI (M: F)
0.334	7.03±5.09	20	5.04±4.28	13	Age of Abnormal MRI
0.488	12:8	20	9:4	13	Gender of Abnormal MRI (M: F)

**Table 2:** MRI findings and abnormalities in two groups

	Disorder			Intractable		Drug-Responsive			P-value
1	NL			21 (29.16%)		184 (92.46%)			<0.001*
2	Neuronal migration disorder			7 (9.72%)		0			<0.001*
3	Hippocampal abnormality	Hippocampal atrophy	Lft	2	Total=10(13.88%)	1		Total=1 (0.05%)	<0.001*
			Rt	1		0			
			Bilateral	2		0			
		Hippocampal sclerosis	Lft	2		0			
			Rt	1		0			
			Bilateral	1		0			
		Hippocampal rounding and medial displacement		1		0			
4	Focal lesion	Probably mass lesion		8	Total=10 (13.88%)	1		Total=1 (0.05%)	<0.001*
		Probably focal dysplasia		2		0			
5	lat. ventricle temporal Dilatation	Rt		2	Total= 3 (4.16%)	1		Total= 2 (1.00%)	0.118
		Lf		0		1			
		Bilateral		1		0			
6	Hypogenesis of corpus callosum			2 (2.77%)		1 (0.05%)			0.173
7	Developmental cyst			2 (2.77%)		0			0.069
8	Hydrocephalus			1 (0.05%)		0			0.265
9	Porencephalic cyst			3 (4.16%)		0			0.018*
10	Brain malacia			1 (0.05%)		0			0.265
11	Gliosis			3 (4.16%)		0			0.018*
12	atrophy	Global		4	Total= 8 (11.11%)	2	Total= 4 (2.01%)		0.003*
		Lft hemisphere		2		1			
		Cerebellar		2		1			
13	Hyper signal intensities			1 (0.05%)		6 (3.01%)			0.679
14	Total			72		199			-

## In Conclusion

Demographic variables such as age and gender have no significant relationship with the occurrence of intractable epilepsy or medical responsive epilepsy. The MRI findings, including hippocampal abnormalities, focal lesions, porencephalic cysts, gliosis, and brain atrophies, have a significant relationship with the occurrence of intractable epilepsies. Moreover, neuronal migration disorder can be concurrent with intractable epilepsies.

## Author’s Contribution

Azime Khosronejad: substantial contributions to the conception and drafting the work

Elham Rahimian: MRI interpretation and neuroradiologic consultation

Mohammad Raiszadeh: neurosurgery consultation

Shahriar Najafizade: Acquisition,analysis

A. Ranaie-kenarsari: Acquisition and analysis of data and interpretation of data for the work

S.Amirsalari: clinical diagnosis ,patient selection for LTM,EEG interpretation and final decisions

## Conflicts of Interest

None
